# The Effects of Ultraviolet A/B Treatments on Anthocyanin Accumulation and Gene Expression in Dark-Purple Tea Cultivar ‘Ziyan’ (*Camellia sinensis*)

**DOI:** 10.3390/molecules25020354

**Published:** 2020-01-15

**Authors:** Wei Li, Liqiang Tan, Yao Zou, Xiaoqin Tan, Jiacheng Huang, Wei Chen, Qian Tang

**Affiliations:** College of Horticulture, Sichuan Agricultural University, Chengdu 611130, China; lw816816@163.com (W.L.); tlq615@163.com (L.T.); zouyao82@163.com (Y.Z.); Xqintan@163.com (X.T.); sicau_hjc@163.com (J.H.); chenwei2551@163.com (W.C.)

**Keywords:** UV-A, UV-B, *Camellia sinensis*, Ziyan, anthocyanin biosynthesis, transcriptome

## Abstract

‘Ziyan’ is a novel anthocyanin-rich tea cultivar with dark purple young shoots. However, how its anthocyanin accumulation is affected by environmental factors, such as ultraviolet (UV), remains unclear. In this study, we observed that UV light treatments stimulated anthocyanin accumulation in ‘Ziyan’ leaves, and we further analyzed the underlying mechanisms at gene expression and enzyme activity levels. In addition, the catechins and chlorophyll contents of young shoots under different light treatments were also changed. The results showed that the contents of total anthocyanins and three major anthocyanin molecules, i.e., delphinidin, cyanidin, and pelargonidin, were significantly higher in leaves under UV-A, UV-B, and UV-AB treatments than those under white light treatment alone. However, the total catechins and chlorophyll contents in these purple tea plant leaves displayed the opposite trends. The anthocyanin content was the highest under UV-A treatment, which was higher by about 66% than control. Compared with the white light treatment alone, the enzyme activities of chalcone synthase (CHS), flavonoid 3′,5′-hydroxylase (F3′5′H), and anthocyanidin synthase (ANS) under UV treatments increased significantly, whereas the leucoanthocyanidin reductase (LAR) and anthocyanidin reductase (ANR) activities reduced. There was no significant difference in dihydroflavonol 4-reductase (DFR) activity under all treatments. Comparative transcriptome analyses unveiled that there were 565 differentially expressed genes (DEGs) of 29,648 genes in three pair-wise comparisons (white light versus UV-A, W vs. UV-A; white light versus UV-B, W vs. UV-A; white light versus UV-AB, W vs. UV-AB). The structural genes in anthocyanin pathway such as flavanone 3-hydroxylase (*F3H), F3′5′H, DFR*, and *ANS*, and regulatory gene *TT8* were upregulated under UV-A treatment; *F3′5′H*, *DFR*, *ANS*, and *UFGT* and regulatory genes *EGL1* and *TT2* were upregulated under UV-AB treatment. However, most structural genes involved in phenylpropanoid and flavonoid pathways were downregulated under UV-B treatment compared with control. The expression of *LAR* and *ANR* were repressed in all UV treatments. Our results indicated that UV-A and UV-B radiations can induce anthocyanin accumulation in tea plant ‘Ziyan’ by upregulating the structural and regulatory genes involved in anthocyanin biosynthesis. In addition, UV radiation repressed the expression levels of *LAR*, *ANR*, and *FLS*, resulting in reduced ANR activity and a metabolic flux shift toward anthocyanin biosynthesis.

## 1. Introduction

Tea, produced from the new bud and leaves of *Camellia sinensis* (L.) O. Kuntze, is one of the three most widely consumed nonalcoholic beverages throughout the world [1]. There are six kinds of tea (black tea, green tea, yellow tea, dark tea, white tea, and oolong tea) by using different processing technology in China. It is also an important commercial crop in many countries. Tea contains a large number of secondary metabolites that are beneficial to human beings. It was reported that there were nearly 4000 bioactive compounds in tea and one third was contributed by polyphenols [2,3]. Studies have shown that drinking teas can prevent cancer [4], alleviate oxidative stress, and suppress hyperglycemia and antidiabetic effect [5].

Most commercial tea cultivars have green buds and young leaves, with several exceptions, such as ‘Anji baicha’ (albino), ‘Chuanhuang 1′ (yellow), and ‘Ziyan’ (purple). In recent years, more and more researchers focus on these distinct tea cultivars, because they generally contain higher levels of special bioactive components like amino acids and anthocyanins than green tea cultivars. *Camellia sinensis* ‘Ziyan’ is a novel purple-leaf cultivar that accumulates a large amount of delphinidin-related anthocyanins [6]. The purple-colored buds and leaves are closely associated with anthocyanin accumulations [6,7,8]. Meanwhile, mainly due to the high level of anthocyanin, purple shoot tea was reported to have more pharmacological benefits as compared with ordinary tea. For example, Rashid et al. [9] reported that tea anthocyanins could cross the blood–brain barrier and enhance the brain’s antioxidant capacity in mice.

Environmental factors such as light (quality, intensity, and period), temperature, and drought stress can significantly impact the phenotype of young shoot and leaf of tea plant. Currently, several studies on purple-leaf tea cultivars have been reported, which focused on constituent of anthocyanins, health efficacy, and anthocyanin biosynthesis. As we know, anthocyanin synthesis is one of the downstream branches of the flavonoid biosynthetic pathway. And lots of genes involved in anthocyanin biosynthesis, transportation, and regulation were characterized to be involved in anthocyanin accumulation in Arabidopsis [10]. Catechins are one of the most important components of tea polyphenols. Anthocyanin and catechin biosynthesis share some key enzyme catalytic steps [11]. In the anthocyanin biosynthesis pathway, there are four stages: the phenylpropanoid pathway stage including phenylalanine ammonia-lyase (PAL), cinnamic acid 4-hydroxylase (C4H), and 4-coumaroyl CoA ligase (4CL); the polyketide pathway stage to provide malonyl-CoA; the early biosynthesis stage including chalcone synthase (CHS), chalcone isomerase (CHI), flavanone 3-hydroxylase (F3H), flavonoid 3′-hydroxylase (F3′H), and flavonoid 3′,5′-hydroxylase(F3′5′H); and the later biosynthesis stage mainly involving dihydroflavonol 4-reductase (DFR), anthocyanidin synthase (ANS), and UDP-glucose: flavonoid 3-O-glucosyltransferase (UFGT) [12,13]. After anthocyanidins are glycosylated, methylated, or acylated to form anthocyanins, anthocyanins are transported to the vacuoles, facilitated by glutathione-S-transferase (GST), ATP-binding cassette (ABC), and toxic compound extrusion (MATE) family proteins [14]. Both the structural and regulatory genes involved in anthocyanin biosynthesis have been isolated and identified [15,16,17,18,19,20,21,22,23]. It has been reported that the expression of structural genes usually dependents on the transcriptional activity of MYB-bHLH-WD40 (MBW) ternary complex, which consists of R2R3-myeloblastosis (MYB), bHLH (basic helix–loop–helix), and WD40-repeat proteins [15]. This complex binds to promoters of structural genes of anthocyanin biosynthesis and regulates their expression [16]. MYBs have been well characterized in many plants, such as Arabidopsis [17], apple [18], sweet cherry [19], petunia [20], potato [21], lychee [22], and grape [23]. NAC and WRKY transcription factors have been also reported for regulating anthocyanin accumulation [24,25]. The photoreceptors, including UV RESISTANCE LOCUS 8 (UVR8) for reception of UV-B, phototropins (PHOT1, PHOT2) absorbing ultraviolet-A (UV-A)/blue light, cryptochromes (CRY1, CRY2, CRY3) and phytochromes (PHYA-E) responding to red/far-red light, have been well documented [26]. ELONGATED HYPOCOTYL 5 (HY5) is required for the transcriptional activation of the *PFG1/MYB12* and *PFG3/MYB111* genes under UV-B and visible light and is also a direct target of CONSTITUTIVE PHOTOMORPHOGENIC 1 (COP1) and has been linked to the activation of structural genes and transcription factors in the flavonoid pathway in response to light [27,28]. In addition, some PHYTOCHROME INTERACTING FACTORs (PIFs) family members could interact with phytochromes [29] and participate in anthocyanin biosynthesis by binding the promoters of anthocyanin biosynthesis-related genes [30].

The anthocyanin biosynthesis in plants is regulated by light and light quality, such as ultraviolet A (UV-A), ultraviolet B (UV-B), blue and red lights [31]. The previous studies showed that a B-box protein, MdCOL11, is involved in UV-B- and temperature-induced anthocyanin biosynthesis in apple peel [32]. Hydroxycinnamic acids and anthocyanins increased in UV-B-treated samples during apple storage [33]. UV-B and low temperature synergistically enhanced the expression of anthocyanin biosynthetic genes, especially *MdCHS*, *MdANS*, *pUFGluT* [34], and *MdMYBA* [18], resulting in increased anthocyanin biosynthesis. *CHS*, *DFR*, and *F3H* showed a positive correlation with anthocyanin accumulation in UV-B-irradiated lettuce leaves [35]. UV-B radiation stimulates the expression of genes encoding enzymes involved in the anthocyanin biosynthetic pathway, and increases the accumulation of anthocyanins in Arabidopsis [36]. UV-A induction of anthocyanin accumulation was also observed in eggplant [37], grape [38], carrot cells [39], and Arabidopsis [40]. The UV-A induction of *CHS* expression involved the CRYl photoreceptor [36]. *PAL, CHS, F3H, DFR*, and *ANS* genes were upregulated during a 24-h exposure to UV-A in turnip [41]. In contrast, UV-B irradiation failed to induce *CHS* expression [41]. In addition, the phenylpropanoids in different plant species were found to respond differently to UV-B [42,43,44,45]. And how UV radiation affects anthocyanin accumulation in tea plant leaves at gene expression and enzyme activity levels remains unclear.

Chlorophyll is a lipid-soluble pigment located in the thylakoid membrane. The chlorophyll metabolic pathway in higher plants is well characterized. It consists of three steps: chlorophyll biosynthesis, chlorophyll cycle, and chlorophyll degradation [46]. UV radiation damages photosynthesis by inactivation of photosystems I and II (PS I, PS II) and decrease chlorophyll contents [47]. The previous studies showed that contents of chlorophyll a, b and carotenoids of pepper leaves were reduced significantly in plants exposed to UV-B and UV-C radiation [48]. UV-B irradiation also induced a decrease in chlorophyll and carotenoid concentrations of *Sorghum* [49,50].

Through two consecutive years of observation and determination, we found that the content of anthocyanins in ‘Ziyan’ was higher by >26.19% (unpublished data) in the summer than that in the spring and autumn. As we know, the temperature and ultraviolet intensity are significantly higher in summer than those in spring and autumn. However, the previous studies have shown that high temperature was not required for anthocyanin accumulation. Therefore, we speculated that ultraviolet radiation might be an important environmental factor for promoting anthocyanin accumulation in ‘Ziyan’. The young shoots as economical organs contain a large amount of anthocyanins in ‘Ziyan’ plants. Their young leaves display dark purple color, and they turn into green as these leaves become old. In the present study, we used this special tea cultivar ‘Ziyan’ to explore how UV-A and UV-B affect anthocyanin accumulation. We have applied the high-throughput RNA sequencing (RNA-seq) on ‘Ziyan’ young leaves that were treated with UV-A, UV-B, UV-A + UV-B, and white light, respectively. The expression patterns of differentially expressed genes (DEGs) involved in anthocyanin biosynthesis were analyzed. The study provides a further understanding of UV radiation induction of anthocyanin accumulation in the tea plants, which may guide tea cultivation in tea gardens and scientific research on other anthocyanin-rich plants.

## 2. Results

### 2.1. Color Analysis of the Second Top Leaves

**Figure 1:**
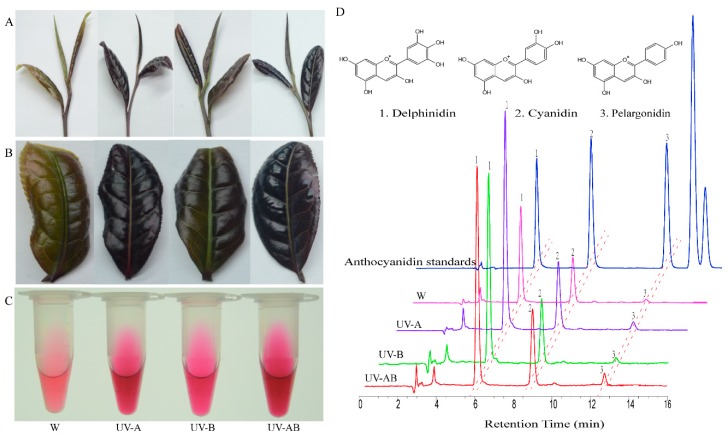
Phenotype of young shoots, anthocyanin extract and component analysis of anthocyanidins in different treatments. (**A**–**C**) represented one bud and two leaves (1B2L), the second top leaf of one bud and two leaves, and anthocyanin extract of one bud and two leaves in W, UV-A, UV-B and UV-AB treatment, respectively. (**D**) High-performance liquid chromatography (HPLC) chromatogram of the pigments extracted from one bud and two leaves in different treatments. W, UV-A, UV-B, and UV-AB represent under white light, ultraviolet-A, ultraviolet-B, and ultraviolet-(A + B) treatment, respectively.

The color of the second top leaf of the shoot tips (including one bud and 1st and 2nd leaves) was measured by a colorimeter and expressed by *L a b* color space values (Table 1). In the control (white light, W), the value of *L*, *b*, and *h°* was highest than ultraviolet (UV) treatments. It indicated that the leaves were brighter and yellower. Leaves presented a darker purple color under ultraviolet-A (UV-A) treatment, which showed the lowest *L*, *b*, *h°* and the highest *a* and *C* values. And it was easy to know that the order for color depth from high to low was UV-A, ultraviolet-(A + B) (UV-AB), ultraviolet-B (UV-B), and white light (W) (Table 1 and Figure 1A–C).

### 2.2. Ultraviolet Light Induce Anthocyanin Accumulation in ‘Ziyan’ Young Shoots

The anthocyanin extract solutions of each sample under different treatment were detected by high-performance liquid chromatography (HPLC) system (Figure 1D). The total content of anthocyanin of three different ultraviolet treatments is significantly higher (*p* < 0.05) than white light treatment (Table 2). And the anthocyanin contents with UV-A treatment were the highest (107.98 mg/100gFW), which were 65.94% higher than control. Delphinidin, cyanidin, and pelargonidin were detected in all of the samples (Figure 1D). The amounts of three pigments in UV-A treatment were higher than other treatments. Compared to W, the three pigment contents in young shoots under ultraviolet treatment were significant increase. Delphinidin, cyanidin, and pelargonidin contents in UV-A treatment increased by 64.57%, 80.12%, and 49.34% than those of control, respectively. These results indicated that UV-A, UV-B, and UV-AB stimulated anthocyanin (including total and constituent content) accumulation in ‘Ziyan’, and UV-A had the strongest promotional effect on anthocyanin accumulation.

### 2.3. Photosynthetic Pigment Content in Young Shoots

**Figure 2:**
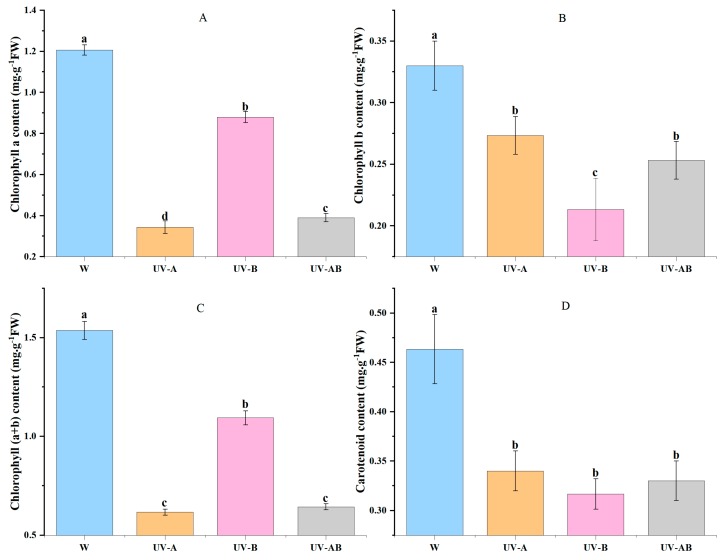
Photosynthetic pigment content in young shoot under different treatments. (**A**) Chlorophyll a contents. (**B**) Chlorophyll b contents. (**C**) Chlorophyll (a + b) contents. (**D**) Carotenoid contents. Error bars indicate MS ± SD of three biological replicates. Different letters among treatments indicate a significant difference at *p* < 0.05 based on the analysis of variance (ANOVA) (Duncan test).

The chlorophyll a contents were very low in UV-A and UV-AB treatments (Figure 2A). The chlorophyll b contents were the lowest in UV-B treatment (Figure 2A). The variation tendency of chlorophyll (a + b) contents was similar to that of chlorophyll a (Figure 2A,C). There was no significant difference in carotenoid contents of three UV treatments. Compared with W, the chlorophyll and carotenoid contents were significantly decreased (>29.22%) in UV treatments (*p* < 0.05) (Figure 2A–D). Combined with anthocyanin content data (Table 1), we found that the higher the anthocyanin contents contained, the lower the photosynthetic pigment contents involved in young shoot. Thus, we hypothesized that UV induced anthocyanin accumulation, which inhibited chlorophyll formation.

### 2.4. Catechins Contents in Shoot Tips of Ziyan

We also measured catechin contents in the shoot tips. Compared with the control, the total and constituent contents of catechins in all of these UV treatments were significantly reduced (Table 3). There was no significant difference in total catechins of the samples between UV-A and UV-AB treatments, but they were significantly lower than those of samples treated with UV-B. Both total catechins content and most of the catechins component contents were the lowest by UV-A irradiation. Epigallocatechin (EGC), epicatechin (EC), epigallocatechin gallate (EGCG), and epicatechin gallate (ECG) accounted for >86.86% of total catechins in all of samples, which were the major catechins components. These results indicated that ultraviolet radiation was not conducive to the accumulation of catechins.

### 2.5. Activities of Main Enzymes in Anthocyanin Biosynthesis

**Figure 3:**
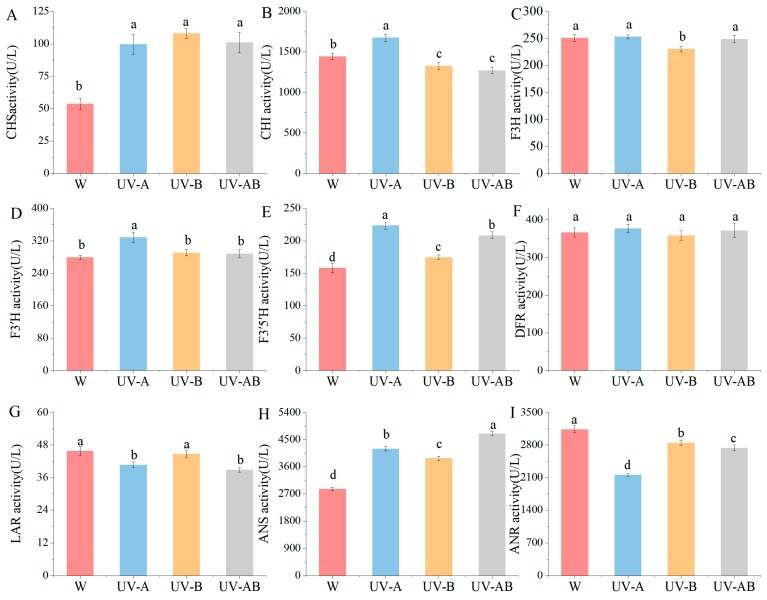
Activities of CHS, CHI, F3H, F3′H, F3′5′H, DFR, LAR, ANS, and ANR (**A**–**I**). The activities of each enzyme in one bud and two leaves of ‘Ziyan’ tea plant under different treatments were detected. CHS, chalcone synthase; CHI, chalcone isomerase; F3H, flavone 3-hydroxylase; F3′H, flavonoid 3′-hydroxylase; F3′5′H, flavonoid 3′,5′-hydroxylase; DFR, dihydroflavonol 4-reductase; LAR, leucoanthocyanidin reductase; ANS, anthocyanidin synthase; ANR, anthocyanidin reductase. Error bars indicate MS ± SD of three biological replicates. Different letters among treatments indicate a significant difference at *p* < 0.05 based on the analysis of variance (ANOVA) (Duncan test).

The anthocyanin biosynthesis pathway was one of flavonoid synthesis pathway. Anthocyanin was synthesized under the catalysis of various enzymes, such as CHI, CHS, F3H, ANS, and so on. In the present study, the activities of different enzymes related to anthocyanin synthesis were significantly affected by different light quality treatments (Figure 3). There was no significant difference in CHS activity among three UV treatments, whereas it was >85.58% higher than control (Figure 3A). The CHI activity was highest in UV-A treatment among all treatments. However, it was significantly lower in UV-B and UV-AB treatments than control (Figure 3B). There was no significant difference in F3H activity in W, UV-A, and UV-AB, but F3H activity in all samples of these three treatments was significantly higher than that in UV-B treatment (Figure 3C). Activity of F3′H in UV-B treatment was higher by 17.52% than W (*p* < 0.05), while there was no significant difference between W, UV-A, and UV-AB treatments (Figure 3D). Activity of F3′5′H was higher in UV irradiation than W by 10.50%, and F3′5′H activity was significantly higher in UV-A treatment than in other treatment (Figure 3E). The DFR activity was not significantly different in all samples (Figure 3F, *p* > 0.05). The leucoanthocyanidin reductase (LAR) activity displayed no significant difference in UV-B compared with the control, but it was significantly higher than that in UV-A and UV-AB treatments (Figure 3G). ANS activity in samples under UV-A, UV-B, and UV-AB radiation was 47.38%, 37.78%, and 64.73% higher than that in W, respectively (Figure 3H, *p* < 0.05). Compared with W, the activities of ANR were significantly decreased by 30.77%, 7.97%, and 11.47% in samples under UV-A, UV-B, and UV-AB treatments, respectively (Figure 3I, *p* < 0.05). These results indicated that UV-A/B treatment promoted the enzymatic activity of anthocyanin biosynthesis and decreased the activity of ANR that catalyzed anthocyanidin reduction.

### 2.6. Transcriptome Sequencing and Gene Mapping

To elucidate the molecular mechanism of anthocyanin accumulation in ‘Ziyan’ young shoot underlying UV treatment, we used the RNA-Seq high-throughput sequencing technology. cDNA libraries of eight samples (W_1, W_2, UV-A_1, UV-A_2, UV-B_1, UV-B_2, UV-AB_1, and UV-AB_2, two biological repeats) were sequenced. A total of 59.63 Gb clean bases were obtained by Illumina Hiseq 2500 platform. GC content and Q30 bases were more than 45.14% and 92.11%, respectively (Table 4). The mapped ratio of clean reads mapped to tea plant reference genome was 62.62%–65.19% (Table 4). A total of 29,648 genes expression was mapped to the genome. In this study, we excavated 6389 new genes of which 5518 genes annotated function based on the comparison results, the genetic structure optimization analysis, and the analysis of variable splicing prediction.

### 2.7. Analysis of Differentially Expressed Genes (DEGs)

**Figure 4:**
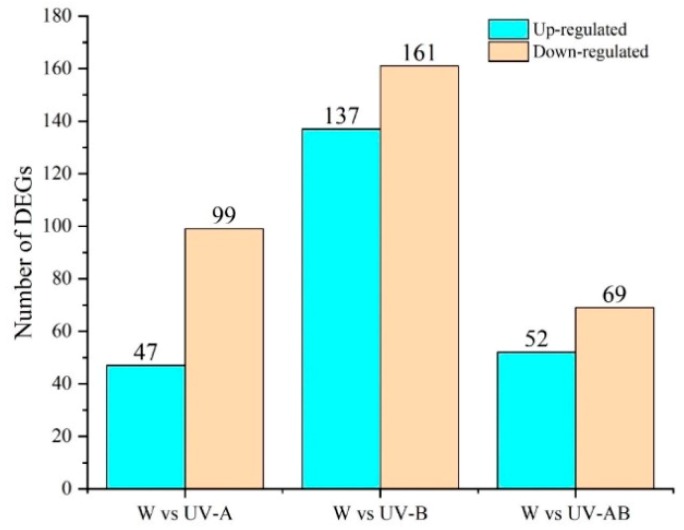
Numbers of differentially expressed genes (DEGs) in W vs. UV-A, W vs. UV-B, and W vs. UV-AB.

To clarify the gene expression in response to ultraviolet, significantly differentially expressed genes were obtained for three ultraviolet treatments compared with control, with DESeq2 software set at FDR < 0.01 and fold change at ≥2.0. In the three comparisons of W vs. UV-A (W vs. UV-A represents up/downregulated genes in UV-A when compared with W (control) treatment), W vs. UV-B and W vs. UV-AB, the DEGs numbers were 146 (47 upregulated, 99 downregulated), 298 (137 upregulated, 161 downregulated) and 121 (52 upregulated, 69 downregulated), respectively (Figure 4). These DEGs indicated that the genes in response to light quality were different in young shoots. And all of these DEGs had been annotated against public databases including Clusters of Orthologous Groups of proteins (COG), Gene Ontology (GO), Kyoto Encyclopedia of Genes and Genomes (KEGG), Eukaryotic Orthologous Groups (KOG), Non-redundant protein sequences (Nr), Homologous protein family (Pfam), Swiss: non-redundant protein sequence (Swiss-Prot), and evolutionary genealogy of genes: Non-supervised Orthologous Groups (eggNOG) (Table S1).

### 2.8. GO and KEGG Enrichment Analysis of DEGs

**Figure 5:**
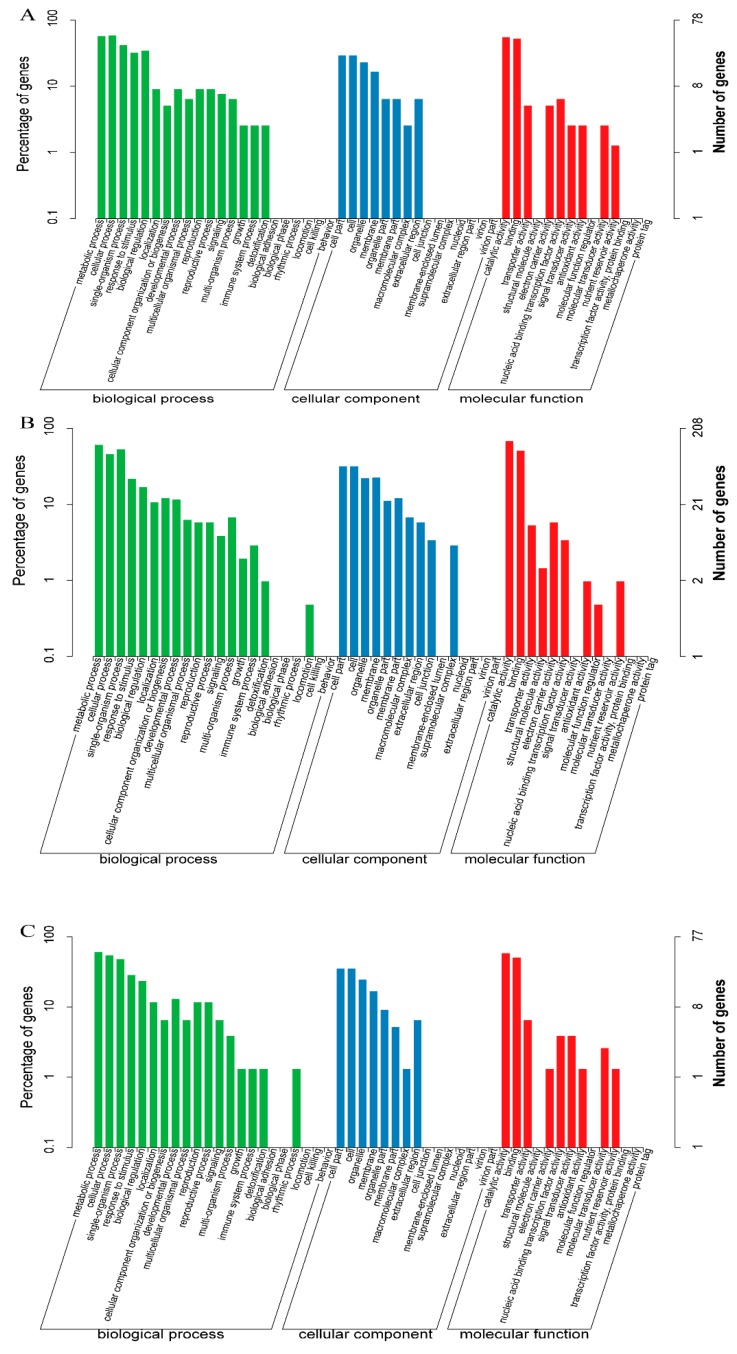
Gene Ontology (GO) enrichment analysis in pairwise comparisons. (**A**) GO enrichment analysis in W vs. UV-A. (**B**) GO enrichment analysis in W vs. UV-B. (**C**) GO enrichment analysis in W vs. UV-AB.

To better know the function of DEGs, Gene Ontology (GO) and Kyoto Encyclopedia of Genes and Genomes (KEGG) enrichment analyses were conducted. DEGs of W vs. UVA were analyzed by using Blast-GO, and the results were shown in Figure 5A (Table S2). All the DEGs were successfully assigned to three categories including biological process, cellular component, and molecular function with 33 groups. In the biological process, DEGs were assigned to 16 groups and the major DEGs were assigned into “metabolic process” (GO: 0008152), “cellular process” (GO: 0009987), “single-organism process” (GO: 0044699), “response to stimulus” (GO: 0050896), and “biological regulation” (GO: 0065007). Within the cellular component category, DEGs were assigned to 8 subcategories and many DEGs were classified in “cell part” (GO: 0044464), “cell” (GO: 0005623), “organelle” (GO: 0043226), and “membrane” (GO: 0016020). The molecular function category including 9 subcategories and the subcategories of “catalytic activity” (GO: 0003824) and “binding” (GO: 0005488) were mostly related. The DEGs assigned into GO terms in W vs. UV-B and W vs. UV-AB were similar to those in W vs. UV-A group (Figure 5B,C; Tables S3 and S4).

**Figure 6:**
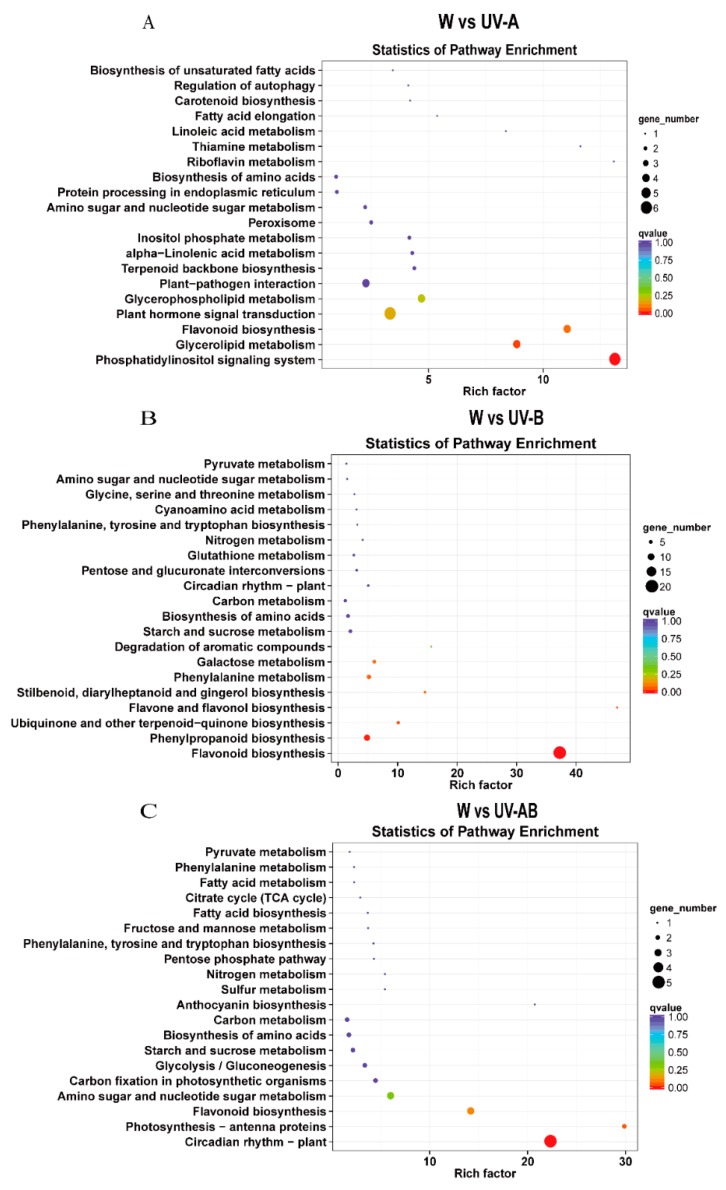
Kyoto Encyclopedia of Genes and Genomes (KEGG) enrichment analysis in pairwise comparisons. (**A**) KEGG enrichment analysis in W vs. UV-A. (**B**) KEGG enrichment analysis in W vs. UV-B. (**C**) KEGG enrichment analysis in W vs. UV-AB.

Furthermore, all the DEGs in the W vs. UV-A group, W vs. UV-B group, and W vs. UV-AB group were matched against the KEGG pathway database, respectively. In the W vs. UV-A group, DEGs assigned 31 pathways. The main pathways were plant hormone signal transduction, phosphatidylinositol signaling system, flavonoid biosynthesis, glycerolipid metabolism, and glycerophospholipid metabolism (Figure 6A; Table S5). All DEGs were assigned 54 pathways in the W vs. UV-B group. The majority of genes were found in flavonoid biosynthesis and phenylpropanoid biosynthesis (Figure 6B; Table S6). The DEGs in W vs. UV-AB group were significantly enriched in the circadian rhythm-plant, amino sugar and nucleotide sugar metabolism, flavonoid biosynthesis and photosynthesis-antenna proteins pathway (Figure 6C; Table S7).

### 2.9. Different Expression Profiles of Genes Associated with Anthocyanin Biosynthesis

**Figure 7:**
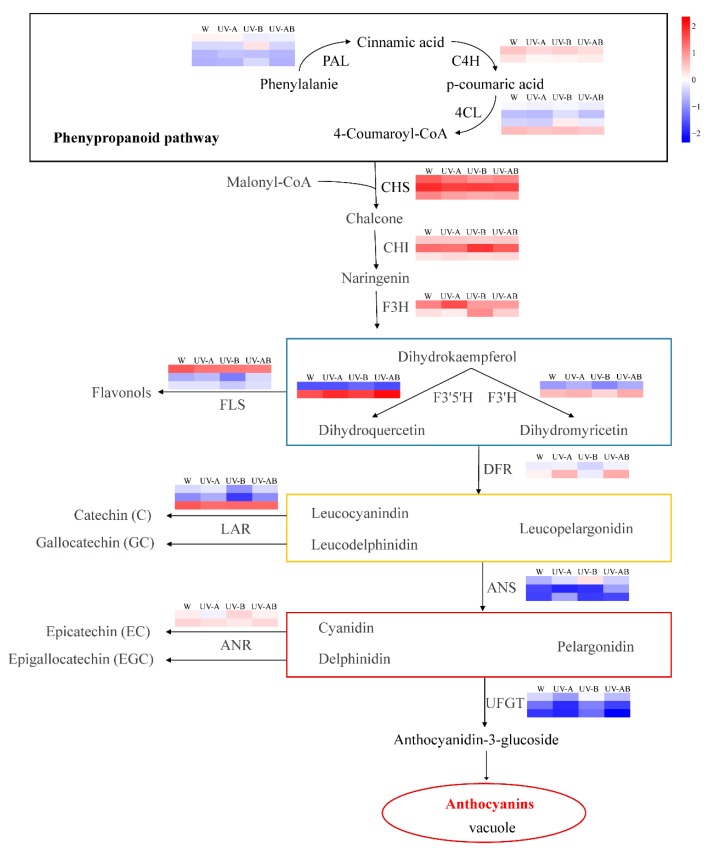
Expression patterns of the genes involved in anthocyanin biosynthesis in different treatments. The color scale at the right represents the normalized value of log_10_ (FPKM), and FPKM (fragments per kilobase of transcript per million mapped reads) values obtained from RNA-Seq data. The expression levels for each gene are shown by colors ranging from low (blue) to high (red).

**Figure 8:**
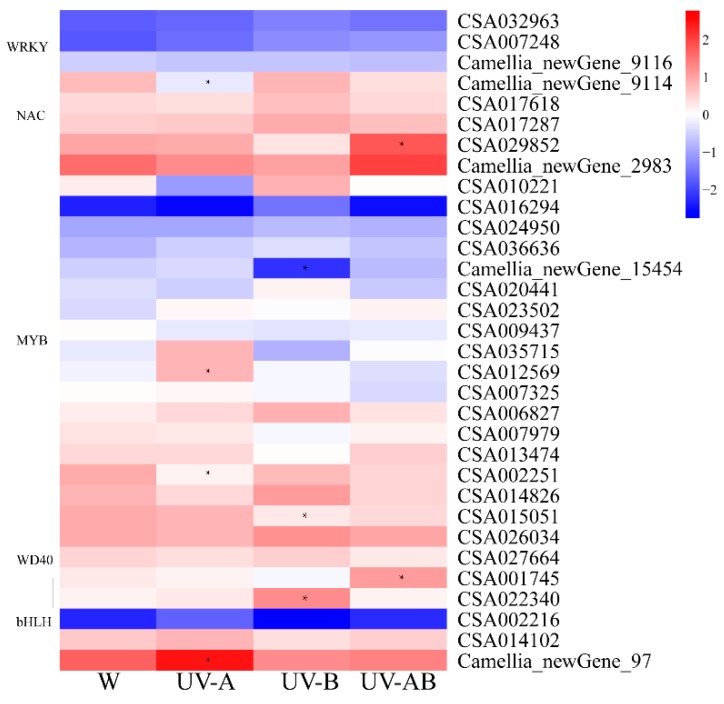
Expression patterns of transcription factors involved in anthocyanin biosynthesis. Asterisk (*) presents gene significantly up- or downregulated in UV-A, UV-B, and UV-AB treatment compared with W. The color scale at the right represents the normalized value of log10 (FPKM), and FPKM (fragments per kilobase of transcript per million mapped reads) values obtained from RNA-Seq data. The expression levels for each gene are shown by colors ranging from low (blue) to high (red).

All DEGs identified in pairwise comparisons were used to identify the candidate genes related to anthocyanin biosynthesis. We investigated the expression levels of 38 transcripts related to the 14 genes encoding enzymes involved in flavonoid biosynthetic pathway (Figure 7). DEG involved in phenylpropanoid pathway was not assigned in the W vs. UV-A group. However, the early biosynthesis contained two structural genes (*F3H* and *F3′5′H*), and the later biosynthesis contained two, structural genes (*DFR* and *ANS*) were contained in flavonoid pathway, as well as four regulatory genes (*TRANSPARENT TESTA 8* (*TT8*), *MYB4*, *WRKY41*, and *TT2*) were involved in regulation of anthocyanin biosynthesis in that group (Figure 7 and Figure 8). Most of the DEGs involved in phenylpropanoid and flavonoid pathways were decreased in W vs. UV-B. One *PAL*, two *C4Hs*, one *4CL*, one *CHI*, three *CHSs*, one *F3H*, one *F3′H*, three *FLSs*, three *LARs*, and one *ANR* were downregulated in UV-B treatment compared to white treatment (Figure 7). From Figure 7 we knew that low expression levels of *FLS, LAR*, and *ANR* were beneficial to anthocyanin accumulation. In addition, one regulatory gene of *bHLH* family, *bHLH3* was upregulated in W vs. UV-B. Two regulatory genes of *MYB* family including positive regulatory TF, *MYB110*, and negative regulatory TF, *C1*, were downregulated in UV-B treatment compared to the white light treatment control (Figure 8). When compared to white light treatment, one DEG (*F3′5′H*) in the early biosynthesis and two DEGs (*DFR* and *ANS*) in the later biosynthesis were upregulated and one *UFGT* contained in anthocyanin pathway was downregulated in UV-AB treatment (Figure 7). Moreover, two activation regulatory genes (*EGL1* and *TT2*) showed a higher expression level in W vs. UV-AB group (Figure 8, Table S8). Based on these results, we found that the effect of UV irradiation with different wavelengths on inducing anthocyanin accumulation in ‘Ziyan’ leaves was different. The ability to induce anthocyanin accumulation in ‘Ziyan’ leaves was stronger by UV-A or UV-AB than UV-B.

### 2.10. Transcription Factors (TFs) Analysis

The ternary complex MBW consisting of a MYB, a bHLH, and a WD40-repeat acts as a critical transcriptional machinery regulating anthocyanin biosynthesis. In the present study, twenty *MYBs*, five *bHLHs*, four *WRKYs*, two *NACs*, and one *WD40*, which may be involved in flavonoid and anthocyanin biosynthesis, were identified (Figure 8). The activation transcription factors *TT8* (Camellia_newGene_97) and *TT2* (CSA012569) were significantly upregulated, and the negative TF, *MYB4*, was markedly downregulated in W vs. UV-A (Figure 8). The *bHLH3* (CSA022340) was markedly upregulated, whereas *C1* (CSA015051) and *MYB110* (Camellia_newGene_15454) were prominently downregulated in W vs. UV-B (Figure 8). The *EGL1* (CSA001745) and *TT2* (CSA029852) genes were notably upregulated in W vs. UV-AB group (Figure 8). In addition, the other two transcription factor families *WRKY* and *NAC* have also been demonstrated to be involved in anthocyanin biosynthesis. However, all of these *WRKYs* and *NACs* displayed no significant difference between each treatment except one *WRKY* in W vs. UV-A group. Compared with white light control, the expression levels of these *MYBs*, *bHLHs*, *WD40*, *NACs*, and *WRKYs*, except several TFs genes mentioned above, did not up/downregulated by UV treatment (Figure 8). Based on these results, we speculated that these positive and negative regulatory TFs may play an important role in regulation of anthocyanin biosynthesis in tea plants under UV radiation.

### 2.11. Expression Patterns of the Genes Involved in Chlorophyll Metabolism

**Figure 9:**
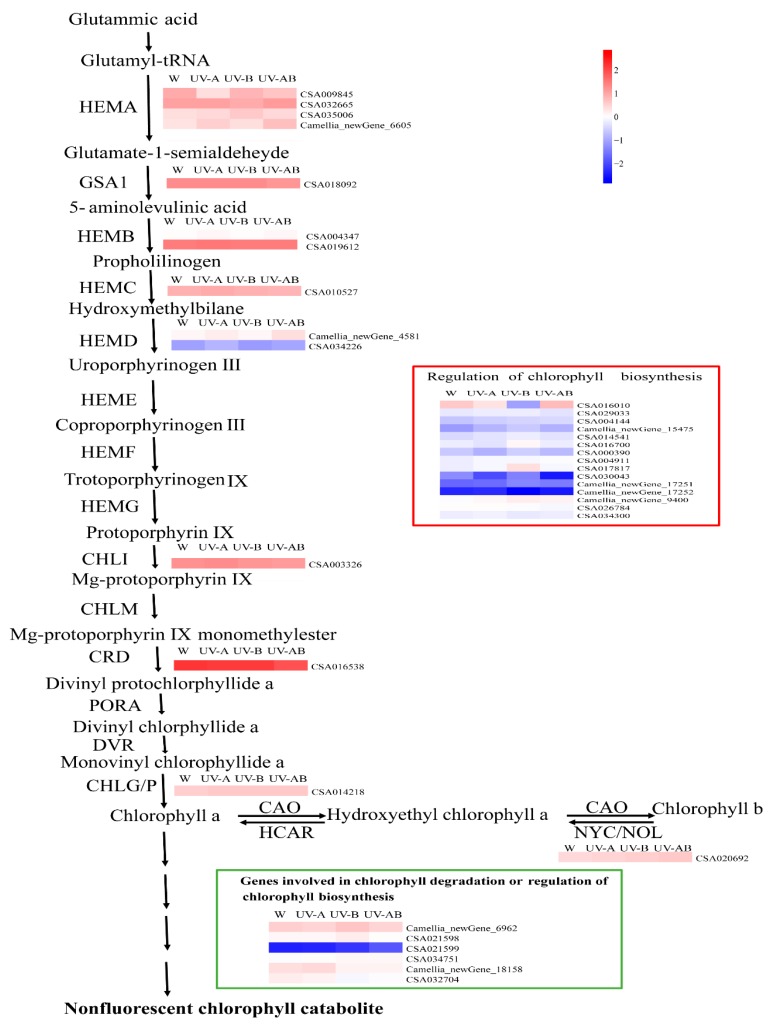
Expression patterns of the genes related to chlorophyll metabolism in each treatment. The color scale at the right represents the normalized value of log10 (FPKM). Genes in the red box are the genes involved in regulation of chlorophyll biosynthesis, and genes in the green box are the genes involved in regulation of chlorophyll degradation.

In the three pairwise comparisons (W vs. UV-A, W vs. UV-B, and W vs. UV-AB), majority of genes involved in chlorophyll biosynthesis showed a lower level by UV light treatments than by white light treatment. Notably, gene *ELIP1* (CSA016010) in W vs. UV-AB group was significantly decreased (Figure 9 and Table S9). The results indicated that UV irradiation did not promote chlorophyll biosynthesis. None of the genes involved in chlorophyll cycle and degradation was found to be DEG in W vs. UV-A, W vs. UV-B, and W vs. UV-AB comparisons (Figure 9). All the regulatory genes of chlorophyll biosynthesis had a low-level expression except CSA016010 in each treatment. On the contrary, almost all the expression levels of genes involved in chlorophyll degradation were higher.

### 2.12. Light Signal Perception and Transduction Component

**Figure 10:**
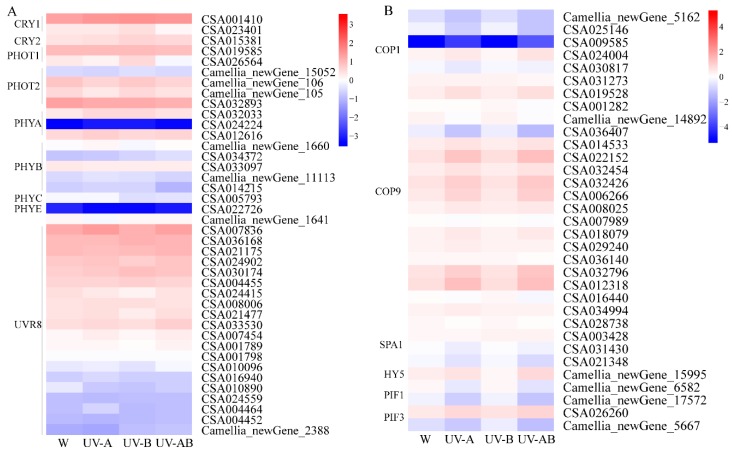
Expression patterns of genes involved in light signal perception (**A**) and transduction (**B**). The color scale at the right represents the normalized value of log10 (FPKM), and FPKM (fragments per kilobase of transcript per million mapped reads) values obtained from RNA-Seq data. The expression levels for each gene are shown by colors ranging from low (blue) to high (red).

Four types of photoreceptors, including phytochromes (PHYs), phototropins (PHOTs), cryptochromes (CRYs), and UV resistance locus 8 (UVR8), have been reported to respond to different light qualities. We identified all of these photoreceptor genes by using gene function annotation. The expression level of almost all of these photoreceptors in UV treatments was lower than those in white light treatment control (Figure 10). However, only one gene *PHOT1* (CSA026564) was significantly downregulated in UV-AB treatment compared to white light treatment (Figure 10A). The expression level of *COP9*, a light signal transduction repressor, was higher than those of other repressor genes *COP1* and *SPAs* (Figure 10B). These light signal transduction repressors showed different expression patterns under W, UV-A, UV-B, and UV-AB treatments. *COP9* showed the highest expression in UV-A and UV-AB, respectively. For the *SPA* family, the expression level of *SPA1* increased, but *SPA3* transcript level decreased in UV treatment. *HY5* as a positive regulator of the light signaling pathway exhibited a higher expression level in UV-AB treatment than in other treatments. Compared with white light control, UV-B treatment increased *PIF1* and *PIF3* transcript levels.

### 2.13. Quantitative Real-Time PCR (qRT-PCR) Validation of the Gene Expression Patterns

To further confirm the accuracy and reliability of the gene expression patterns from transcriptomic data, 20 genes were selected and validated by qRT-PCR. The results showed that the variations of all selected genes were similar to those in RNA-Seq data (Figure S1), which indicated that the transcriptome data were reliable.

## 3. Discussion

The anthocyanin biosynthesis pathway in higher plants is well understood, and anthocyanin synthesis is easily affected by environmental factors such as light (including light quality, light intensity, and photoperiod), temperature, and water. Light quality, especially UV radiation and some other specific light qualities (e.g., red and blue light), could regulate anthocyanin biosynthesis in fruits and induce accumulation of anthocyanin, which have been reported in previous studies [51,52,53,54]. However, most of the previous studies focused on the effect of UV on anthocyanin content and one or several genes or enzyme activities in anthocyanin synthesis pathway. In this study, anthocyanin, catechin, and chlorophyll contents of young shoots via different light qualities were analyzed. Transcriptome analyses clarified the gene expression pattern involved in anthocyanin biosynthesis and revealed the molecular mechanism of accumulation of anthocyanin under UV radiation. In addition, it also helped to identify the key genes related to anthocyanin biosynthesis in tea plant.

### 3.1. Anthocyanin, Catechin, and Photosynthetic Pigment Contents Variations with UV Treatment

Leaf color is generally correlated with pigment content, component ratio, and metabolism. Leaves appear red, blue, pink, and purple may be due to anthocyanin content and proportion in pigments, while green leaf generally relates to chlorophyll content and its ratio in pigment [55,56]. In this study, the contents of total anthocyanins, anthocyanin components marked differences in different light qualities (Table 2). Anthocyanin contents in all of the UV treatments were significantly higher than those in white light treatment. In previous studies, UV-B has been shown to increase anthocyanin levels [51,57,58], and UV-A has also been showed to promote anthocyanin accumulation [41,59]. In our study, the result indicated that the effect of anthocyanin accumulation induced by different UV light was different. UV-A induced the increase of anthocyanin content more effectively than UV-B and UV-AB. Anthocyanin contents under UV-A, UV-B, and UV-AB treatment were 65.94%, 23.61%, and 39.67% higher than those of white light treatment, respectively. This may be due to several structural and regulatory genes upregulated in anthocyanin biosynthesis while *LAR* and *ANR* genes downregulated under UV treatments. In addition, F3′H, F3′5′H, and ANS activity under UV treatments were higher than those of control, while ANR activity showed the opposite trend. In the *L a b* color space, a lower *L*, *b*, and *h°* and a higher *a* and *c* value indicated that the leaf color was deeper purple in young shoots by UV light irradiation than white light treatment (Figure 1A,B).

Catechins belong to flavan-3-ols, and they account for 12–24% of dry weight mass of teas. They are also the main components of tea polyphenols. Catechins biosynthesis pathway was branched from flavonoid pathway. Catechin and anthocyanin biosynthesis shared several key catalytic enzymes in the flavonoid pathway (Figure 7). Leucoanthocyanidins are converted by leucoanthocyanidin reductase (LAR) into catechin (C) and gallocatechin (GC). Anthocyanidins can be catalyzed by anthocyanidin reductase (ANR) to form epicatechin (EC) and epigallocatechin (EGC) (Figure 7). To better understand how UV stimulated anthocyanin accumulation, we examined catechins content. In the present study, both total and constituent contents of catechins were significantly reduced in UV treatment compared with white light (Table 3). Catechins contents decreased by UV-A, UV-B, and UV-AB treatments by 15.68%, 6.88%, and 13.61%, respectively, compared to those of control. We speculated that UV radiation might inhibit the activity of enzymes involved in catechin synthesis and result in a decrease of catechin content. For instance, the activity of ANR was reduced significantly in UV treatment compared with white light (Figure 3). Thus, we speculated that this may be due to the inhibition of enzymes involved in catechins synthesis, resulting in a shift in metabolic fluxes toward anthocyanin biosynthesis rather than catechins synthesis.

Chlorophyll is an important pigment in leaves. The anthocyanin/chlorophyll ratio is related to leaf color. The anthocyanin content gradually decreased, and the chlorophyll content slowly increased as the leaf developed in the tea plant [60] and Ornamental Kale [61]. UV-B radiation can significantly reduce chlorophyll content in *Capsicum annuum* [48,62] and Sorghum leaves [49]. There was no effect on chlorophyll content in *Capsicum annuum* [48] and cocoplum leaves [63] by UV-A treatment. However, in the present study, chlorophyll content was significantly decreased by UV radiation, especially by UV-A, by 59.74%. The results also showed the higher the anthocyanin content, the lower the chlorophyll content in young shoots (Figure 2), which indicated that chlorophyll and anthocyanin levels were negatively correlated. Meanwhile, these results indicated that UV-A has different effects on chlorophyll content of different plants. And the previous studies showed that anthocyanin content increased and chlorophyll content decreased in *Capsicum annuum* [48] and Sorghum leaves [49] by UV-B treatment. These results are consistent with ours. Thus, we hypothesized that UV-induced accumulation of anthocyanin may inhibit the accumulation of chlorophyll or UV irradiation suppressed chlorophyll biosynthesis.

### 3.2. Activity of Enzyme and DEGs Involved in Anthocyanin Biosynthesis

UV-A/B treatment increased the accumulation of anthocyanins by stimulating the expression of many genes encoding enzymes involved in the anthocyanin biosynthetic pathway [34,64]. Phenylalanine ammonia-lyase (PAL) performs a crucial function in the phenylpropanoid biosynthesis pathway and accelerates anthocyanin synthesis [65]. UV-B and UV-A treatments stimulated PAL activity and regulated *PAL* expression in maize [66] and tomato [59], respectively. In this study, however, UV-A and UV-AB treatments did not stimulate the expression of genes (including *PAL*, *4CL*, and *C4H*) involved in phenylpropanoid pathway. On the contrary, the expression levels of *PAL* (CSA016076), *4CL* (CSA007753), and *C4H* (CSA022101 and CSA032295) were significantly downregulated by UV-B treatment compared with control (Figure 7). The anthocyanin pathway genes *F3H* (CSA004930), *F3′5′H* (CSA031792)*, DFR* (Camellia_newGene_7155), and *ANS* (CSA035767) were significantly upregulated under UV-A treatment compared with white light control. *F3′5′H* (CSA031792)*, DFR* (Camellia_newGene_7155), and *ANS* (CSA021078) were also significantly upregulated in W vs. UV-AB. *F3H* (CSA004930) and *ANS* (CSA011508) were significantly upregulated, while *CHS* (CSA024718, CSA029707 and CSA029775), *CHI* (CSA008262), and *DFR* (Camellia_newGene_7155 and Camellia_newGene_5799) were significantly downregulated by UV-B compared with white light control. All of these genes mentioned above were positively correlated with anthocyanin biosynthesis [67]. *FLS* (CSA003707, CSA006950 and CSA008358), *LAR* (CSA014151, CSA014943 and CSA018523), and *ANR* (CSA011986) were significantly downregulated in W vs. UV-B, which were consistent with the reduced flavonol and catechins biosynthesis in the treatment. The lower the activity of LAR and ANR, the weaker the competition ability to utilize leucoanthocyanidins and anthocyanidins for catechins synthesis. Thus, metabolic fluxes might be shifted to anthocyanin biosynthesis and accumulation. UV radiation markedly promoted the activities of CHS, F3′5′H, and ANS (Figure 3). These enzymes may be the most important enzymes to catalyze anthocyanin biosynthesis via UV induction in ‘Ziyan’ tea leaves.

*TRANSPARENT TESTA 2* (TT2), an R2R3-MYB transcription factor (TF) [26], and TT8, a bHLH TF [68], have been reported to participate in the regulation of anthocyanin biosynthesis. A previous study showed that there was a negative correlation between *AtMYB4* expression and *C4H* expression under UV-B radiation [69]. In this study, UV-A stimulated the expression of the positive TF genes *TT8* and *TT2* but inhibited the expression of the negative regulatory TF *MYB4*. *bHLH3*, *EGL1*, and *TT2* that regulated anthocyanin accumulation [70,71,72] were upregulated by UV-B and UV-AB radiation, respectively. Whereas *C1* and *MYB110* were downregulated by UV-B. *C1* repressed *DFR* and *UFGT* expression levels [73], while *MYB110* appeared to be the activator of anthocyanin biosynthesis in purple kiwifruit [74]. Therefore, the transcription factors mentioned above may be the key TFs to regulate anthocyanin synthesis induced by ultraviolet light in tea plant ‘Ziyan’.

### 3.3. Effect of UV Light on Photoreceptors and Transduction Components

Photoreceptors (PHYA-E) absorb red/far-red light, cryptochromes (CRY1, CRY2, CRY3) and phototropins (PHOT1, PHOT2) sense UV-A/blue light, and a UV-B photoreceptor UV RESISTENCE LOCUS8 (UVR8) primarily responds to UV-B [75]. In the present study, transcriptome analysis further revealed the changes in photoreceptors genes at the transcriptional level in tea plants under UV irradiation. We found that gene *PHOT1* (CSA026564) was significantly downregulated in UV-AB compared with white light control. A previous study also reported that the expression level of *PHOT1* remarkably decreased under UV light [76]. The expression patterns of other photoreceptors including CRY1, CRY2, PHYA, PHYB, PHYC, and PHYE were not significantly different under UV irradiation compared with white light treatment control. Transcriptional levels of all UVR8 genes were similar among these treatments and were not further upregulated by UV-B irradiation. A possible explanation is that changes in expression levels of photoreceptors may have occurred earlier than sample collection since light induction of gene expression change can be detected within hours [77]. 

### 3.4. The Expression Pattern of Genes Involve in Chlorophyll Biosynthesis

*HEMA1* is involved in the early steps of chlorophyll biosynthesis [61]. *HEMA1* loss-of-function mutants showed completely yellow color and did not thrive under normal growth conditions [78]. In the present study, the expression level of *HEMA1* (CSA009845) was the lowest by UV-A treatment. Most genes involved in chlorophyll biosynthesis under UV irradiation were lower in expression than those under white light treatment control. The chlorophyll contents were lower under UV treatments, which were in agreement with the expression patterns of genes involved in chlorophyll biosynthesis. Thus, our results indicated that UV irradiation did not induce expression levels of chlorophyll biosynthesis genes. Compared with white light treatment control, the chlorophyll content of ‘Ziyan’ leaves decreased by ultraviolet light treatment. However, none of the genes involved in chlorophyll cycle and degradation was found to be DEG in the three pairwise comparisons (W vs. UV-A, W vs. UV-B, and W vs. UV-AB), suggesting UV irradiation mainly impaired chlorophyll synthesis to different degrees.

## 4. Materials and Methods

### 4.1. Plant Materials and Ultraviolet Treatments

One-year-old purple tea cultivar ‘Ziyan’ (*Camellia sinensis* (L.) O. Kuntze cv. Ziyan) was used in this study. The healthy plants with similar size were transplanted to the plastic pots (length:width:height = 45 cm:30 cm:25 cm). Four pots were prepared and each pot contained 36 plants arranged in three rows, representing three replicates. Subsequently, the tea plants were clipped to approximately 25 cm height. The cultural conditions in growth chamber (Shanghai Sanfa Technology Co., Ltd., Shanghai, China) maintained a photoperiod of 14/10 h (light/dark) at 25/18 °C (light/dark) and approximately 80% relative humidity. The light quality treatments were as follows: (i) white light (W) as control, (ii) white light +UV-A (UV-A), (iii) white light +UV-B (UV-B), and (iv) white light +UV-A + UV-B (UV-AB). White light intensity was 200 μmol m^−2^s^−1^ photon flux densities in (i)–(iv). The radiation intensity of UV-A in treatment (ii) and UV-B in treatment (iii) were 600 μW·cm^−2^. In treatment (iv), both UV-A and UV-B radiation intensity were 300 μW·cm^−2^. UV-A radiation was provided by a special lamp (PHILIPS TL-D 18W, Pila, Poland) with a characteristic peak at 365 nm. UV-B radiation was provided by a special lamp (PHILIPS NARROWBAND TL 20W, Hamburg, Germany) with a characteristic peak at 311 nm. White light provided by light-emitting diode (LED) lamp. White light of all treatment exposure time was at 8:00 to 22:00, and UV radiation time was two periods 8:00–10:00 and 16:00–18:00, respectively. The samples (one bud and two leaves, 1B2L) were separately harvested in each treatment when the majority of young shoots became 1B2L. The collected samples were divided into two portions, one portion was used for RNA extraction and another portion was used for pigments, catechins, and enzyme activity assays. The samples of two portions were immediately frozen in liquid nitrogen and then stored at −80 °C. There were two independent biological replicates for RNA-seq analysis and three independent biological replicates for pigment, catechins, enzyme activity, and quantitative real-time PCR (qRT-PCR) assays.

### 4.2. Color Measurement

Color of the second top leaf of one bud and two leaves was measured by a CM-2600d spectrophotometer (Konica Minolta, Tokyo, Japan). Each leaf was measured 5 times avoiding the main vein. The parameters of the instrument chosen a D65 illuminant and a 45°/normal illuminating. The color space data of *L*, *a*, *b*, *C* (chroma), and *h°* (hue angle) values were recorded. *L* refers to brightness to darkness (*L* > 0 for bright; *L* < 0 for dark), *a* refers to intensity in greenness-redness (*a* > 0 for red; *a* < 0 for green), *b* refers to intensity in blueness -yellowness (b > 0 for yellow; b < 0 for blue), and *C* indicates color saturation.

### 4.3. Anthocyanin Content Measurement by HPLC Method

Fresh leaves were harvested from ‘Ziyan’ tea plants under the experiments and rapidly frozen in liquid nitrogen. After grinding the frozen leaf samples into a fine powder in liquid nitrogen, approximately 0.2 g of powder was taken and soaked in 4 mL of methanol containing 1% (*v*/*v*) hydrochloric acid for 2 h at 4 °C. After centrifugation at 5000 rpm for 10 min, the supernatant was transferred into a brown volumetric flask. Then, the residues were extracted twice again in the same way, but extraction buffer was 3 mL and soaked for 2 h and 12 h, respectively. After centrifugation at 5000 rpm for 10 min, all supernatants were combined into a brown volumetric flask. The acid hydrolysis HPLC method was applied with slight modification [79]. Briefly, 0.2 mL of the extract was mixed with 0.4 mL 5 mol·L^−1^HCl in a 1.5 mL tube. The tube was placed in a preheated dry bath at 90 °C for 30 min, and the hydrolyzed samples were immediately cooled in an ice bath. An Agilent 1260 HPLC system (Agilent, Palo Alto, CA, USA) and Titank-C18 column (250 mm × 4.6 mm, 5 μm, Phenomenex Inc., Los Angeles, CA, USA) were used for the HPLC measurement. The eluents were mobile phase A (water:acetonitrile:formic acid = 87:3:10, *v*/*v*/*v*) and mobile phase B (100% acetonitrile). The chromatographic conditions were as follows: 0 min, 15% B and 30 min, 30% B. The injection volume was 10 μL, the flow rate was 1 mL·min^−1^, and the C18 column was maintained at 35 °C. Anthocyanins were detected at 520 nm by an Agilent VWD detector. 5 reference standards (peonidin chloride, pelargonidin chloride, delphinidin chloride malvidin chloride, and cyanidin chloride) were purchased from Chroma Dex (Los Angeles, CA, USA).

### 4.4. Measurement of Photosynthetic Pigment Content

A 0.2 g fresh sample was used for pigment extraction. For the photosynthetic pigment measurements, the samples were homogenized with 3 mL 95% ethanol, a little of the quartz sand, and calcium carbonate, and then 10 mL 95% ethanol was added to grind the tissue white and let sit for 5 min. Subsequently, the homogenate was filtered to the brown volumetric flask, and the residue was rinsed repeatedly with 95% ethanol, finally, filtrate brought to a final volume of 25 mL [80]. Sample absorbance at 470 nm, 649 nm, and 665 nm was measured by a UV spectrophotometer (T5, Sumimit Instrument, Shanghai, China). The following formulas were used to calculate the pigment content:

Chlorophyll a content (Ca) = 13.95A_665 nm_ − 6.88A_649 nm_ (1)

Chlorophyll b content (Cb) = 24.96A_649 nm_ − 7.32A_665 nm_ (2)

Carotenoids content (Cx·c) = (1000A_470_ − 2.05C_a_ − 114.8C_b_)/245 (3)

### 4.5. Catechins Measurement by HPLC Method

Catechins were extracted from one bud and two leaves as described by Chinese National Standard GB/T8313-2018. The HPLC was run on an Agilent 1260 system (Agilent, Palo Alto, CA, USA) using Titank-C18 column (250 mm × 4.6 mm, 5 μm, Phenomenex Inc., Los Angeles, CA, USA). Mobile phase A contained 9% acetonitrile and 2% acetic acid with 20 μg/mL EDTA, and mobile phase B contained 80% acetonitrile and 2% acetic acid with 20 μg/mL EDTA. The elution conditions were as follows: 100% A for 8 min, a linear gradient to 68% A in 15 min, and then holding for 10 min. The C, EC, GC, EGC, ECG, EGCG, GCG, and CG reference standards were purchased from Sigma (Saint Louis, MO, USA). The HPLC conditions were as follows: 10 μL of sample injection volume, flow rate of 1 mL/min, column temperature of 35 °C, and detection wavelength of 278 nm.

### 4.6. Measurement of Anthocyanin Biosynthesis-Related Enzymes Activity 

The main enzymes (CHS, CHI, F3H, F3′H, F3′5′H, DFR, LAR, ANS, and ANR) activity was measured by corresponding plant ELISA kits (Shanghai Fusheng Industry Co., Ltd., Shanghai, China). Sample (0.5 g) was ground into a fine powder in liquid nitrogen, and 4.5 mL phosphate-buffered saline (PBS, pH 7.4, 0.01 mol/L) was added to the powder. The mixture centrifuged at 5000 rpm for 15 min at 4 °C. The supernatant was transferred into the tube for enzyme activity assays and then measured according to the manufacturer’s recommendations. Briefly, (a) all reagents were balanced at room temperature for 20 min before starting the assay. (b) 10 μL of testing sample and 40 μL of sample diluent were added into the same microplate well. (c) 100 μL of horseradish peroxidase (HRP)-conjugate reagent was added to each well. Then, the microplate was immediately sealed with an adhesive strip and incubated for 60 min at 37 °C. (d) The liquid in microplate wells was discarded, and the microplate was patted dry with absorbent papers. Then the microplate wells were washed with washing solution (400 μL). The microplate was let stand for 1 min and then the microplate was patted dry on an absorbent paper, and the washing process was repeated for five washes. (e) 50 μL of chromogen solution A and 50 μL chromogen solution B were added to each well on the microplate. Then, the microplate was gently mixed and incubated immediately at 37 °C in dark for 15 min. (f) 50 μL of stop solution was added to each well and the optical density value was recorded at 450 nm in a microplate reader within 15 min.

### 4.7. RNA Extraction, Library Construction, and RNA-Seq

Total RNA of each sample was extracted according to the instruction manual of the TRIzol Reagent (Invitrogen, CA, USA). RNA concentration was measured using NanoDrop 2000 (Thermo Fisher Scientific, Waltham, MA, USA). RNA integrity was assessed using the RNA Nano 6000 Assay Kit of the Agilent Bioanalyzer 2100 system (Agilent Technologies, Palo Alto, CA, USA). RNA purity was checked using the NanoPhotometer spectrophotometer (IMPLEN, Los Angeles, CA, USA).

Two samples respectively taken from W, UV-A, UV-B, and UV-AB treatments were used to construct 8 cDNA libraries, which were designated W_1, W_2, UV-A_1, UV-A_2, UV-B_1, UV-B_2, UV-AB_1, and UV-AB_2. A total amount of 1.5 μg RNA per sample was used as input material for the RNA sample preparations. Sequencing libraries were generated using NEBNext UltraTM RNA Library Prep Kit for Illumina (NEB, Ipswich, MA, USA) following the manufacturer’s recommendations, and index codes were added to attribute sequences to each sample. Briefly, mRNA was purified from total RNA using poly-T oligo-attached magnetic beads. Fragmentation was carried out using divalent cations under elevated temperature in NEBNext First Strand Synthesis Reaction Buffer (5X). First-strand cDNA was synthesized using random hexamer primer and M-MuLV Reverse Transcriptase. Second-strand cDNA synthesis was subsequently performed using DNA Polymerase I and RNase H. Remaining overhangs were converted into blunt ends via exonuclease/polymerase activities. After adenylation of 3′ ends of DNA fragments, NEBNext Adaptor with hairpin loop structure were ligated to prepare for hybridization. In order to select cDNA fragments of preferentially 240 bp in length, the library fragments were purified with AMPure XP system (Beckman Coulter, Beverly, MA, USA). Then 3 μL USER Enzyme (NEB, USA) was used with size-selected, adaptor-ligated cDNA at 37 °C for 15 min followed by 5 min at 95 °C before PCR. Then, PCR was performed with Phusion High-Fidelity DNA polymerase, Universal PCR primers, and Index (X) Primer. At last, PCR products were purified (AMPure XP system, Beckman Coulter, Brea, CA, USA) and library quality was assessed on the Agilent Bioanalyzer 2100 system. The clustering of the index-coded samples was performed on a cBot Cluster Generation System using TruSeq PE Cluster Kit v4-cBot-HS (Illumia) according to the manufacturer’s instructions. After cluster generation, the library preparations were sequenced on an Illumina Hiseq 4000 platform and paired-end reads were generated.

### 4.8. Transcriptome Analysis Using Reference Genome-Based Reads Mapping

Low quality reads, such as only adaptor, unknown nucleotides >5%, or Q20 < 20% (percentage of sequences with sequencing error rates <1%), were removed by Perl script. The clean reads that were filtered from the raw reads were mapped to *Camellia sinensis* reference genome (http://www.plantkingdomgdb.com/tea_tree/) using Tophat2 software [81]. The aligned records from the aligners in BAM/SAM format were further examined to remove potential duplicate molecules. Gene expression levels were estimated using FPKM values (fragments per kilobase of exon per million fragments mapped) by the Cufflinks software [82].

### 4.9. Identification of Differentially Expressed Genes (DEGs)

DESeq2 [83] and Q-value were employed and used to evaluate differential gene expression of W vs. UV-A, W vs. UV-B, and W vs. UV-AB. After that, gene abundance differences between those samples were calculated based on the ratio of the FPKM values. The false discovery rate (FDR) control method was used to identify the threshold of the *p*-value in multiple tests in order to compute the significance of the differences. Here, only gene with an absolute value of log2 ratio ≥1 and FDR significance score <0.01 were used for subsequent analysis.

### 4.10. GO and KEGG Enrichment Analysis of DEGs

Gene Ontology (GO) enrichment analysis of the differentially expressed genes (DEGs) was implemented by the GOseq R packages based Wallenius non-central hypergeometric distribution [84], which can adjust for gene length bias in DEGs. KEGG [85] is a database resource for understanding high-level functions and utilities of the biological system, such as the cell, the organism, and the ecosystem, from molecular-level information, especially large-scale molecular datasets generated by genome sequencing and other high-throughput experimental technologies (http://www.genome.jp/kegg/). We used KOBAS [86] software to test the statistical enrichment of differential expression of genes in KEGG pathways.

### 4.11. Gene Expression Validation by qRT-PCR

qRT-PCR was used to verify the reliability of the gene expression patterns obtained by RNA-Seq on the CFX96 real-time PCR system (Bio-Rad, Irvine, CA, USA). There were three independent biological replicates in each treatment. A PrimeScript™ RT enzyme with a gDNA eraser (Takara, Kyoto, Japan) was used for cDNA synthesis according to the manufacturer’s instructions. Gene-specific primers in this step were listed in Supplementary Table S10. qRT-PCR was performed using SYBR Premix (Takara, Kyoto, Japan) according to the manufacturer’s protocols. The polypyrimidine tract-binding protein (*CsPTB*) gene was used as an internal control to normalize the raw data [87]. And the relative expression levels were calculated using the 2^−ΔΔ*C*t^ method [88].

### 4.12. Statistical Analysis

Statistical analyses were performed using the SPSS 20 program (SPSS Inc., Chicago, IL, USA). All data were presented as means ± standard deviation (SD, *n* = 3). The data were subjected to an analysis of variance, and the significant difference test was evaluated using Duncan’s multiple range test.

## 5. Conclusions

In the present study, we found that UV-A, UV-B, and UV-AB light could effectively promote the accumulation of anthocyanins in a purple foliage tea plant variety ‘Ziyan’. Our data showed that the promotion effect of UV-A was strongest. Anthocyanin contents under UV-A, UV-B, and UV-AB treatment were 65.94%, 23.61%, and 39.67% higher than those of white light treatment, respectively. Meanwhile, UV-A, UV-B, and UV-AB treatments reduced the accumulation of chlorophyll and catechins. Catechins contents significantly decreased by UV-A, UV-B, and UV-AB treatments by 15.68%, 6.88%, and 13.61%, respectively, compared to those of control. Chlorophyll content was significantly decreased by UV radiation, especially by UV-A, by 59.74%. Transcriptome analysis showed that the DEGs involved in anthocyanin biosynthesis under UV irradiation play an important role in accumulation of anthocyanin. The effects of UV-A and UV-B irradiation on activity of enzyme involved in anthocyanin biosynthesis were different, and CHS, F3′5′H, and ANS activities were significantly higher in UV treatments than those of under the white light treatment. Collectively, our results indicated that both UV-A and UV-B irradiation promoted the accumulation of anthocyanins by enhancing the expression of several regulatory and structural genes and promoting the activity of some enzymes involved in anthocyanin biosynthesis.

## Figures and Tables

**Table 1:** Color value of tea leaves.

Treatments	*L*	*a*	*b*	*C*	*h°*
W	26.82 ± 0.46 ^a^	1.32 ± 0.03 ^c^	0.93 ± 0.07 ^a^	1.61 ± 0.06 ^b^	35.29 ± 1.40 ^a^
UV-A	22.89 ± 0.58 ^d^	1.89 ± 0.06 ^a^	0.56 ± 0.06 ^c^	1.97 ± 0.07 ^a^	16.60 ± 1.08 ^c^
UV-B	24.98 ± 0.42 ^b^	1.52 ± 0.07 ^b^	0.68 ± 0.04 ^c^	1.66 ± 0.07 ^b^	23.99 ± 1.03 ^b^
UV-AB	23.79 ± 0.35 ^c^	1.79 ± 0.03 ^a^	0.61 ± 0.03 ^c^	1.89 ± 0.03 ^a^	18.91 ± 0.59 ^c^

Note: The different letters following the numbers indicate significant differences between different treatments (*p* < 0.05).

**Table 2:** Anthocyanidin contents in different treatments detected by HPLC method (mg/100 g, Means ± SD).

Treatments	Delphinidin	Cyanidin	Pelargonidin	Total Content
W	50.53 ± 1.01 ^d^	11.92 ± 0.43 ^c^	2.29 ± 0.14 ^b^	65.07 ± 2.02 ^d^
UV-A	83.10 ± 2.36 ^a^	21.47 ± 1.34 ^a^	3.42 ± 0.24 ^a^	107.98 ± 3.43 ^a^
UV-B	60.69 ± 4.13 ^c^	18.22 ± 1.36 ^b^	2.18 ± 0.61 ^b^	80.43 ± 2.07 ^c^
UV-AB	67.82 ± 4.81 ^b^	20.25 ± 1.95 ^a,b^	2.82 ± 0.59 ^a,b^	90.88 ± 5.85 ^b^

Note: The different letters following the numbers indicate significant differences between different treatments (*p* < 0.05).

**Table 3:** Contents of catechins in each treatment (%, dry weight).

	W	UV-A	UV-B	UV-AB
GC	0.47 ± 0.01 ^a,b^	0.44 ± 0.01 ^b,c^	0.50 ± 0.03 ^a^	0.43 ± 0.018 ^c^
EGC	3.84 ± 0.12 ^a^	2.99 ± 0.15 ^c^	3.43 ± 0.08 ^b^	3.23 ± 0.09 ^b^
C	0.30 ± 0.01 ^a^	0.22 ± 0.01 ^c^	0.27 ± 0.01 ^b^	0.14 ± 0.01 ^d^
EC	0.89 ± 0.05 ^a^	0.61 ± 0.03 ^c^	0.41 ± 0.01 ^d^	0.73 ± 0.01 ^b^
EGCG	5.24 ± 0.10 ^a^	4.51 ± 0.08 ^b^	4.66 ± 0.04 ^b^	4.30 ± 0.12 ^c^
GCG	0.10 ± 0.01 ^a^	0.06 ± 0.01 ^c^	0.10 ± 0.01 ^a^	0.08 ± 0.01 ^b^
ECG	1.35 ± 0.01 ^a^	1.18 ± 0.04 ^b^	1.34 ± 0.05 ^a^	1.20 ± 0.02 ^b^
CG	0.18 ± 0.01 ^d^	0.23 ± 0.02 ^c^	0.29 ± 0.01 ^b^	0.34 ± 0.02 ^a^
total	12.12 ± 0.26 ^a^	10.22 ± 0.13 ^c^	11.34 ± 0.05 ^b^	10.47 ± 0.18 ^c^

Note: The different letters following the numbers indicate significant differences between different treatments (*p* < 0.05). GC, gallocatechin; EGC, epigallocatechin; C, catechin; EC, epicatechin; EGCG, epigallocatechin gallate; GCG, gallocatechin gallate; ECG, Epicatechin gallate; CG, Catechin gallate.

**Table 4:** Statistics of Illumina reads and comparison with tea pant genome *.

Samples	Clean Reads	Mapped Reads	Mapped Ratio	Clean Bases	GC Content	% ≥Q30
W_1	24,865,772	15,888,028	63.90%	7,410,633,190	45.42%	92.75%
W_2	27,109,093	17,437,532	64.32%	8,104,781,906	45.70%	92.54%
UV-A_1	25,724,765	16,770,006	65.19%	7,671,009,520	45.64%	92.77%
UV-A_2	23,783,560	15,097,109	63.48%	7,107,164,686	45.47%	92.14%
UV-B_1	22,624,538	14,310,230	63.25%	6,764,209,148	45.14%	92.60%
UV-B_2	25,195,952	15,777,097	62.62%	7,533,165,362	45.41%	92.11%
UV-AB_1	26,123,991	16,732,571	64.05%	7,819,599,246	45.50%	92.19%
UV-AB_2	24,129,091	15,531,463	64.37%	7,220,302,692	46.02%	92.18%

* Tea plant genome: http://www.plantkingdomgdb.com/tea_tree/. W_1, W_2; UV-A_1, UV-A_2; UV-B_1 UV-B_2; UV-AB_1, UV-AB_2 represent two biological replicates for each treatment, respectively.

## Supplementary Materials

Supplementary Materials are available online, Table S1: Summary of functional annotations for DEGs. Table S2: GO analysis of DEGs in W vs. UV-A. Table S3: GO analysis of DEGs in W vs. UV-B. Table S4: GO analysis of DEGs in W vs. UV-AB. Table S5: KEGG enrichment analysis of DEGs in W vs. UV-A. Table S6: KEGG enrichment analysis of DEGs in W vs. UV-B. Table S7: KEGG enrichment analysis of DEGs in W vs. UV-AB. Table S8: Transcription data for transcription factors (TFs) under different treatments. Table S9: Expression patterns of genes involved in chlorophyll metabolism. Table S10: List of primers used for qRT-PCR analysis. Figure S1: qRT-PCR validation of selected genes. The left y-axis shows the relative gene expression levels, and the right y-axis indicates the corresponding expression levels in RNA-Seq data. Data are represented as means ± standard deviation.
